# Supplementary material for: The Expression of the Endocannabinoid Receptors CB2 and GPR55 Is Highly Increased during the Progression of Alzheimer’s Disease in AppNL-G-F Knock-In Mice
Source: Biology (Basel). 2023 May 31;12(6):805. doi: 10.3390/biology12060805 (PMC10295339; doi:10.3390/biology12060805)
Supplement: Supplementary file 1 [file biology-12-00805-s001.zip › biology-2315023-supplementary.pdf]

Supplementary Materials

The Expression of the Endocannabinoid Receptors CB2 and GPR55 is Highly Increased during the Progression of Alzheimer's Disease in *App*$^{NL\text{-}G\text{-}F}$ Knock-In Mice

Dina Medina-Vera [1,2], Hongjing Zhao [3,4], Erika Bereczki [4], Cristina Rosell-Valle [1], Makoto Shimozawa[4], Gefei Chen[5], Fernando Rodríguez de Fonseca [1], Per Nilsson [4,†], Simone Tambaro[4,*,†]

Figure S1: Immunoreactivity of GPR55 in the prefrontal cortex of 12-month-old App^{WT} and App$^{NL\text{-}G\text{-}F}$ mice.

Figure S2: Representative figure showing isolated Astrocyte and Microglia cell culture purity.

Figure S3: Negative control images of GPR55 immunostaining in mouse *App*$^{NL\text{-}G\text{-}F}$ primary cell cultures.

Figure S4: Marked Iba1 and GPR55 immunoreactivity were observed in microglia APP cells that were treated for 24h with Aβ42(1μm).

Figure S5: Changes in CB2 and GPR55 fluorescence intensity after Aβ42 treatment versus control non-treated (con).

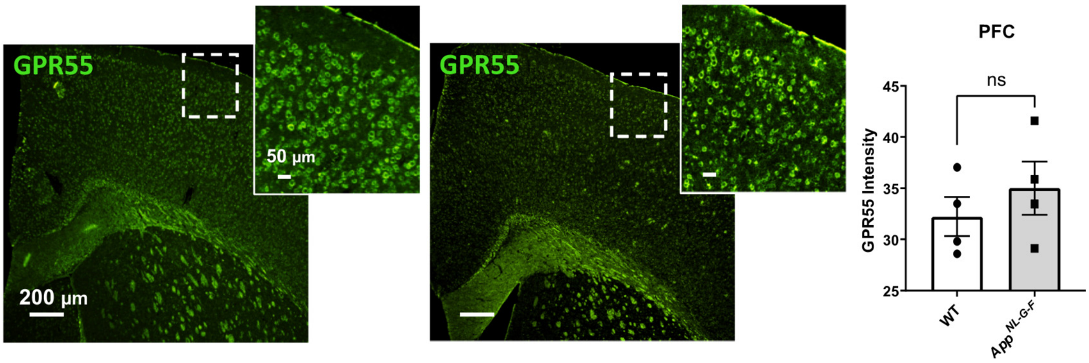

Figure S1: GPR55 expression in the prefrontal cortex of WT and *App*$^{NL\text{-}G\text{-}F}$ mice at 12 months of age. Immunofluorescence images show the widespread distribution of GPR55 receptors (green) in the prefrontal cortex (PFC), revealing no genotype-dependent changes. Scale bars represent 200 μm. Data represent mean ± S.E.M. (*n* = 4 mice per group and 2 sections per mouse were used for quantification). A T-test was performed.

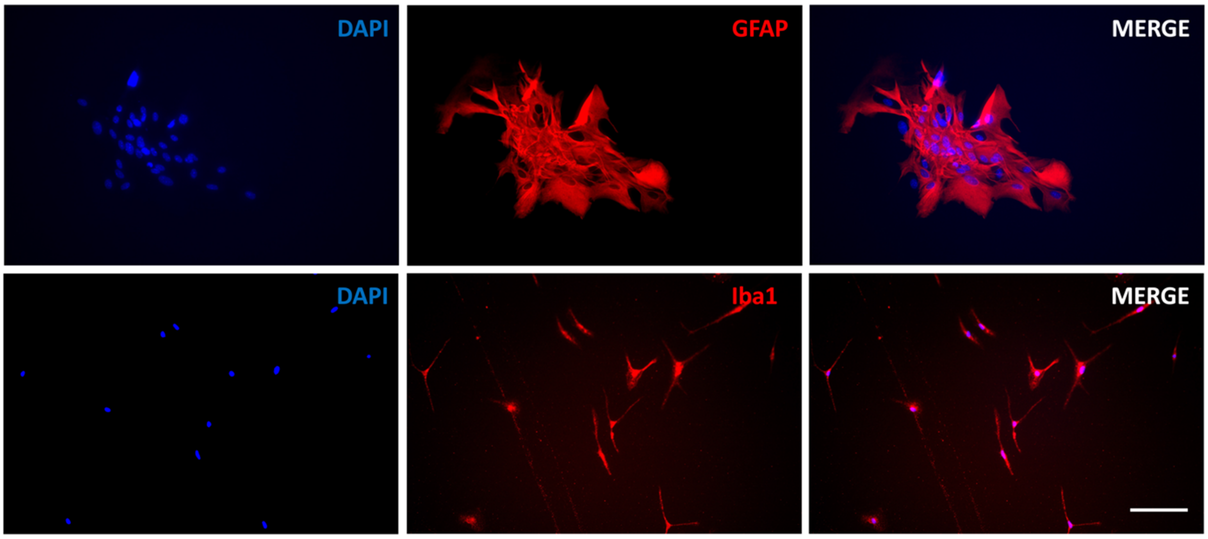

Figure S2: Representative figure showing isolated Astrocyte and Microglia cell culture purity. Mouse WT primary astrocyte cells were isolated by mild trypsinization (0,08% trypsin) from a mixed glia culture and plated in a 24-well plate. The remaining cells were considered microglial cells and were detached using 0.25% trypsin and plated in a 24-well plate. The purity of the two cell preparations was confirmed by the nuclear cell staining (DAPI) merged with the astrocyte-specific marker GFAP for the astrocyte-purify culture and by the merge of the nuclear cell staining DAPI with the microglia-specific marker Iba1 for the microglia-purify culture. Scale bar =100 μm.

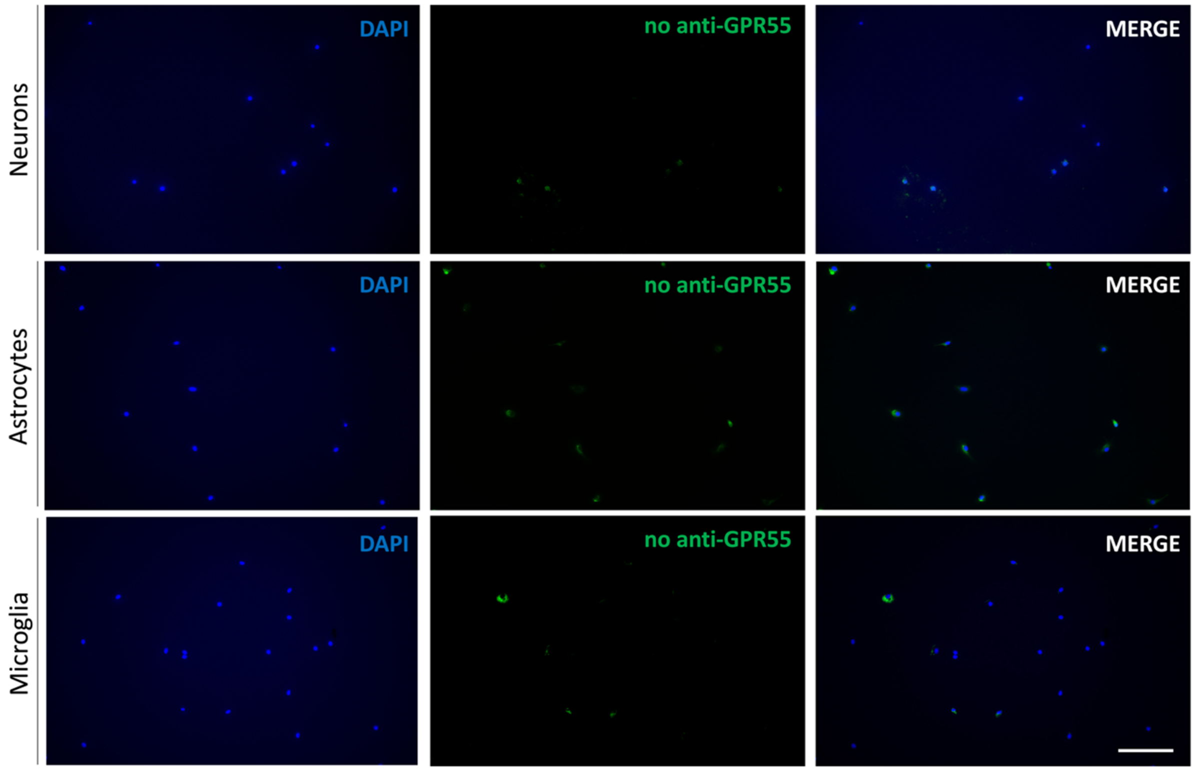

Figure S3: Negative control images of GPR55 immunostaining in mouse *App*$^{NL\text{-}G\text{-}F}$ primary cell cultures. Representative negative control images related to Figure 8, showing the nuclear (DAPI, in blue), omitting GPR55 primary antibody (no anti-GPR55) with the only secondary antibody anti-rabbit AlexaFluor 488 (A32731 Invitrogen, in green), and their combination (Merge) in isolated neurons, astrocytes, and microglia. Scale bar =100 μm.

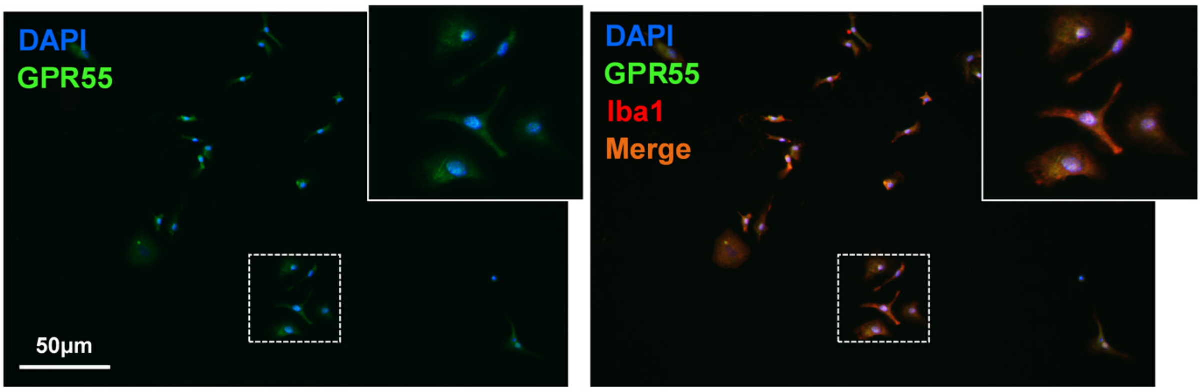

Figure S4: Representative immunostaining of GPR55 in *App*$^{NL\text{-}G\text{-}F}$ microglia after Aβ42 treatment. The cells were treated with Aβ42 1µM for 24 h. The microglia cells were stained with anti-GPR55 (green), DAPI (blue), and anti-Iba1 microglia marker (red). Scale bar =50µm.

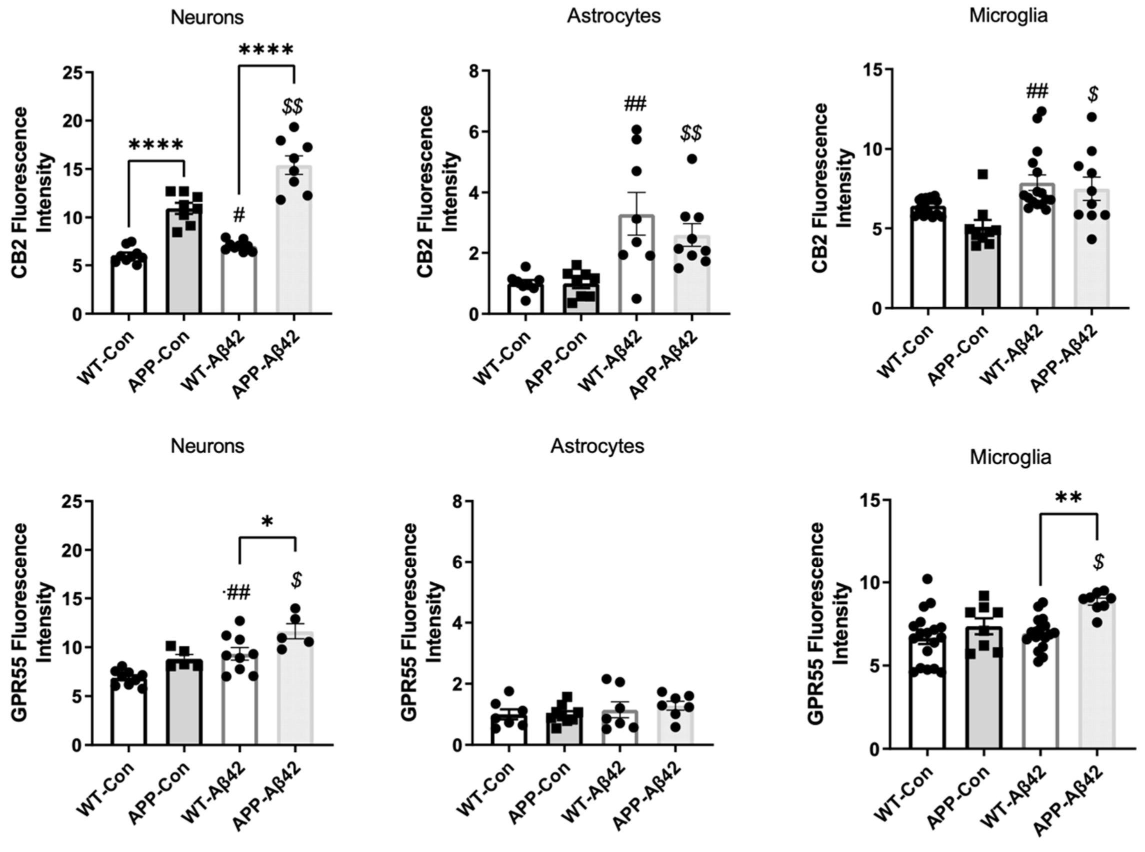

Figure S5: CB2 and GPR55 fluorescence intensity levels in non-treated and Aβ42-treated WT and *App*$^{NL\text{-}G\text{-}F}$ mice primary cell cultures. Graphs are presented as Means ± SEM. Each treatment was performed at least in triplicates, and from each slide, at least 2 images were used for quantification. In the GPR55 staining, in the experimental neuronal group *App*$^{NL\text{-}G\text{-}F}$ treated with Aβ42, one data point was identified and excluded as an outlier. Two-way ANOVA followed by Tukey's multiple comparison tests was used, * denotes $p < 0.05$, ** denotes $p < 0.01$, and **** denotes $p < 0.0001$; # denotes $p < 0.05$ and ## denotes $p < 0.01$ WT-Con vs. WT-Aβ42; $ denotes $p < 0.05$ and $$ denotes $p < 0.01$ APP-Con vs. APP-Aβ42.
